# Intraganglionic Signaling as a Novel Nasal-Meningeal Pathway for TRPA1-Dependent Trigeminovascular Activation by Inhaled Environmental Irritants

**DOI:** 10.1371/journal.pone.0103086

**Published:** 2014-07-31

**Authors:** Phillip Edward Kunkler, Carrie Jo Ballard, Jessica Joan Pellman, LuJuan Zhang, Gerry Stephen Oxford, Joyce Harts Hurley

**Affiliations:** 1 The Department of Biochemistry and Molecular Biology, Stark Neurosciences Research Institute, Indiana University School of Medicine, Indianapolis, Indiana, United States of America; 2 Department of Pharmacology and Toxicology, Stark Neurosciences Research Institute, Indiana University School of Medicine, Indianapolis, Indiana, United States of America; University of Iowa Carver College of Medicine, United States of America

## Abstract

Headache is the most common symptom associated with air pollution, but little is understood about the underlying mechanism. Nasal administration of environmental irritants activates the trigeminovascular system by a TRPA1-dependent process. This report addresses questions about the anatomical pathway involved and the function of TRP channels in this pathway. TRPV1 and TRPA1 are frequently co-localized and interact to modulate function in sensory neurons. We demonstrate here that resiniferatoxin ablation of TRPV1 expressing neurons significantly reduces meningeal blood flow responses to nasal administration of both TRPV1 and TRPA1 agonists. Accordingly resiniferatoxin also significantly reduces TRPV1 and CGRP immunostaining and TRPV1 and TRPA1 message levels in trigeminal ganglia. Sensory neurons of the trigeminal ganglia innervate the nasal epithelium and the meninges, but the mechanism and anatomical route by which nasal administration evokes meningeal vasodilatation is unclear. Double retrograde labeling from the nose and meninges reveals no co-localization of fluorescent label, however nasal and meningeal labeled cells are located in close proximity to each other within the trigeminal ganglion. Our data demonstrate that TRPV1 expressing neurons are important for TRPA1 responses in the nasal-meningeal pathway. Our data also suggest that the nasal-meningeal pathway is not primarily by axon reflex, but may instead result from intraganglionic transmission.

## Introduction

The occurrence of headache in the general population is quite common, yet the triggers for and initiation of headache remains poorly understood. Air pollutants and environmental irritants have been suggested to be one important trigger. For example, increased air pollution is correlated with an increase in emergency room visits for headache symptoms [1]–[3] and headache is one of the most common complaints following human exposure to environmental irritants [4]. The neurobiological link between these chemical triggers and the induction of headache attacks remains unknown.

Headache symptoms are thought to be associated with activation of the trigeminovascular system, i.e., the release of the inflammatory peptides substance P and calcitonin gene-related peptide (CGRP) from trigeminal sensory neurons [5] and subsequent neurogenic inflammation and vasodilatation of meningeal and cerebral blood vessels [6], [7]. Nasal administration of environmental irritants induces meningeal vasodilatation via a CGRP-dependent mechanism [8], but what remains unclear is how the nasal exposure is transduced to a meningeal response. Trigeminal innervation of both the meninges and nasal epithelium is established, but it is not known whether the two share primary afferent neurons. Thus the anatomical substrates linking these apparently distinct, but functionally related trigeminal receptive fields are not obvious. One hypothesis suggests that nasal irritants excite dural afferent neurons via intraganglionic transmission [9], [10]. In this concept, the irritants would first activate receptors on afferents in the nasal mucosa, leading to subsequent release of neurotransmitters from cell soma in the ganglia and resultant paracrine excitation or sensitization of nearby CGRP containing dural afferent neurons. Another putative mechanism includes transmission of the signal from one peripheral site to another site by axon reflex [11], [12].

The transient receptor potential A1 channel (TRPA1) is activated by nasal irritants and thus is a candidate receptor to initiate this headache cascade [8]. TRPA1 is expressed in a subset of CGRP-expressing sensory neurons [13]–[15]. TRPA1 receptors have been shown to act as sensors of many environmental irritants including acrolein [16], formaldehyde [17] and umbellulone [18]. TRPA1 receptors are expressed in trigeminal sensory neurons innervating the nasal epithelium [19] and are postulated to mediate the increased meningeal blood flow observed following intranasal administration of TRPA1 agonists [8]. TRPA1 receptors are highly co-expressed in TRPV1-expressing neurons [13], [20], [21] and these receptors interact functionally [22], which suggests that these neurons and receptors are involved in headache induction. To test this concept, we used the potent TRPV1 agonist resiniferatoxin (RTX) to chemically ablate TRPV1 expressing sensory neurons, presumably including many co-expressing TRPA1. We report here that RTX significantly reduces TRPV1 and CGRP immunoreactivity as well as TRPV1 and TRPA1 mRNA expression in the TG. Furthermore, subsequent irritant-induced dural vasodilatation is significantly reduced following RTX treatment. Using retrograde labeling methods, we experimentally demonstrate the expectation that nasal and cranial blood vessel afferents do not arise from common neurons within the TG. However, neurons from the distinct sensory fields are found in close proximity to each other within the TG. Overall, our results suggest that TRPV1 expressing neurons are important for TRPA1 responses in the nasal-meningeal pathway. These data further suggest that the relevant nasal-meningeal pathway does not involve a peripheral “axon reflex” [11], [12], but may instead reflect intraganglionic transmission.

## Methods

All animal procedures were approved by the Institutional Animal Care and Use Committee at the Indiana University School of Medicine and followed the ethical guidelines of International Association for the Study of Pain [23] Experiments were performed on adult male (170–225 g) Sprague-Dawley rats (Harlan Bioproducts, IN). Rats received a single subcutaneous injection of RTX (200 µg/kg, Tocris Bioscience) under ketamine/xylazine (40 and 5 mg/kg body weight) anesthesia. RTX was dissolved in a mixture of 10% Tween-80 and 10% ethanol in normal saline [24]. Rats in the control group received subcutaneous injection of vehicle only. Rats were returned to the animal facility following recovery until use 1, 2, or 3 weeks after injection.

### Laser Doppler flowmetry

Male rats were anesthetized with ketamine/xylazine (80 and 10 mg/kg body weight, respectively), followed by additional doses of ketamine/xylazine (40 and 5 mg/kg body weight) as needed. Body temperature was maintained at 37°C with a homeothermic blanket. For the measurement of meningeal blood flow, the animals head was fixed in a stereotaxic frame and a cranial window prepared [8], [25] with the dura left intact. Dural blood flow was measured with a laser Doppler flowmeter (TSI, MN). A needle type probe was placed over a large branch of the middle meningeal artery (MMA), distant from visible cortical blood vessels and the cranial window kept moist with synthetic interstitial solution (SIF) consisting of: 135 mM NaCl, 5 mM KCl, 5 mM CaCl_2_, 1 mM MgCl_2_, 10 mM HEPES, 10 mM D-glucose (pH 7.3). Blood flow was sampled at 1 Hz with a Digidata 1320 interface using Axoscope software (Axon Instruments, CA).

### Blood flow drug administration

To stimulate the nasal mucosa, 50 µl of test compound or vehicle solution was applied over a 30 sec period at a site 2 mm into the right nostril using a Pipetman pipette [26]. Solutions of acrolein were prepared fresh daily by diluting in SIF to 30 µM. Stock solutions of capsaicin (10 mM) were dissolved in ethanol and stored at −20°C and then diluted to 100 nM or 1 µM with SIF prior to use. A thirty minute stabilization period preceded all test drug applications to ensure steady basal blood flow measurements. SIF was applied to the nasal mucosa as a control 15 minutes prior to drug application in all experiments and resulted in less than 4% change in basal blood flow in all groups. Most of the animals (28/36) which received acrolein initially were tested with 1 µM capsaicin 15 min later. Thirty minutes after TRP agonist administration, 50 µl of 3 M KCl was administered to the dura to assess the integrity of meningeal blood vessel responses. Following completion of the blood flow experiments, each rat was decapitated and the brain removed. The underlying left TG was rapidly removed, frozen on dry ice and stored at −80°C for later RT-PCR analysis. The right TG was fixed *in situ* by placement of the skull into 4% paraformaldehyde in 0.1 M PBS (pH 7.4) at 4°C overnight for immunocytochemistry.

### Immunocytochemistry

To assess the extent of ablation of TRPV1- or CGRP-positive TG neurons by RTX, immunocytochemistry was performed. Following overnight fixation, the TG was removed from the skull and placed in PBS and then cryoprotected in 10% sucrose for 2 hrs followed by 20% sucrose in PBS overnight at 4°C. Tissues were cut to 35 µm in thickness and collected free-floating in PBS. For TRPV1 immunofluorescent labeling, sections were rinsed in PBS and blocked in 4% normal goat serum and 0.025% Triton-X100 in PBS for 1 hr. The sections were then incubated with the primary antibody (rabbit anti-TRPV1 C-terminus [27]; dilution 1∶7500) diluted in blocking solution overnight at 4°C. Subsequently, sections were rinsed in PBS and incubated with the secondary antibody (Dylight 549-conjugated to goat anti-rabbit IgG, dilution 7.5 µg/ml, Jackson ImmunoResearch) for 1 hr at room temperature. Tissues were processed in a similar manner for CGRP immunofluorescent labeling. Sections were incubated overnight in monoclonal CGRP (1∶2000; Sigma) and for 1 hr in secondary antibody (Alexa Fluor 488 goat-anti-mouse, dilution 7.5 µg/ml, Jackson ImmunoResearch). The sections were rinsed and mounted on slides, dried and treated with Prolong Gold antifade reagent (Life Technologies) before coverslips were affixed. Adjacent tissue sections were also processed with the pan-neuronal marker NeuN (1∶1000; EMD Millipore) to assess total neuronal density following RTX treatment. Fluorescent images were acquired with a 4X objective and cell counts conducted in Photoshop. TG sections containing all 3 trigeminal ganglia subdivisions were used for cell counts. No reactivity was observed in control sections in which primary antibody was omitted.

### RNA isolation and quantitative RT-PCR

RNA from homogenized TG tissue lysate (25–30 mg) was isolated and purified using the RNeasy Mini Kit (Qiagen, Valencia, CA) according to the manufacturer’s instructions. Genomic DNA was removed from isolated RNA with TURBO DNAse (Life Technologies, Foster City, CA) and yield and purity were determined on a Nanodrop ND-1000 Spectrophotometer (Thermoscientific, Franklin, MA). A_260_/A_280_ ratios were between 2.0 and 2.2 for all samples. Single-stranded cDNA was synthesized from 1 µg mRNA using reverse transcriptase (Superscript II reverse transcriptase, Life Technologies) and Oligo(dT)_12–18_ primers (Life Technologies).

Quantitative PCR (qPCR) reactions were run in triplicate on an ABI PRISM 7900HT Sequence Detection System (Life Technologies). The cDNA was amplified for quantitative RT-PCR with SYBR Green PCR Master mix (Life Technologies) and gene specific primers (TRPV1-800 nM, TRPA1-200 nM or β-actin-400 nM). The primers for amplification of rat TRPV1 (Trpv1, Ref NM_031982.1) message were as follows: TRPV1 forward (5′-AGG ACC CAG GCA ACT GTG-3′, T_M_ = 58°C) and TRPV1 reverse (5′-ATC CCT CAG AAG GGG AAC C-3′, T_M_ = 56°C). These primers span exons 15 and 16, align with nucleotides 2456–2474 and 2362–2379 and produce a 113 bp product. The primers for amplification of rat TRPA1 (Trpa1, Ref NM_207608.1) were as follows: TRPA1 forward (5′-GCC CCT GTC TCT GTA AAT AAC C-3′, T_M_ = 55°C) and TRPA1 reverse (5′-CTT GTG TCG CTG ATG TCT TG-3′, T_M_ = 54°C). These primers span exons 11 and 12, align with nucleotides 1276–1297 and 1402–1421 and yield a 146 bp product. The primers for β-actin (Actb, Ref NM_031144.2) were as follows: β-Actin forward (5′-CAC TTT CTA CAA TGA GCT GCG-3′, T_M_ = 54°C) and β-actin reverse (5′-CTG GAT GGC TAC GTA CAT GG-3′, T_M_ = 55°C). The primers span exons 4 and 5, align with nucleotides 345–365 and 473–492 and yield a 148 bp product. A mixture of cDNA template, SYBR Green Master mix and forward and reverse primers was treated with uracil N-glycosylase (Life Technologies) before undergoing the following protocol: 50°C for 2 min, 95°C for 10 min, then 45 cycles of 95°C for 15 s, 60°C for 1 min, followed by 1 cycle of 95°C for 15 s, 60°C for 15 s, and 95°C for 15 s. The PCR products were analyzed with ABI PRISM sequence detection software. The specificity of these amplifications was verified by melt curve analysis with detection of only a single peak. Reactions containing no reverse transcriptase or no template were also run as additional controls.

Real-time quantitative PCR data was analyzed with the ΔΔC_T_ method as described by Livak and Schmittgen (2001) [28]. TRPA1 or TRPV1 transcript levels were compared in TG of saline- and RTX-injected animals at three time points (one, two and three weeks after injection). TRPV1 or TRPA1 values were normalized to β-actin and calibrated to saline injected control data using the ΔΔC_T_ method. β-actin was used as a reference gene and its level was not altered across these experimental conditions. The quantification cycle (Cq) was defined as the number of cycles required to attain a fluorescence threshold of 0.2 units.

### Retrograde labeling

Labeling of the trigeminal innervation of the middle cerebral artery (MCA) followed the procedure of O’Connor and van der Kooy (1986) [29]. Briefly, male rats were anesthetized with ketamine/xylazine (80 and 10 mg/kg body weight, respectively) and a cranial window prepared. The dura was cut and reflected away to expose the right MCA and a small piece of parafilm was positioned underneath the artery. Gelfoam soaked in a 10% solution of 1,1′-dioctadecyl-3,3,3′,3′-tetramethylindocarbocyanine perchlorate (DiI; Life Technologies) dissolved in ethanol was placed on top of the artery after which another piece of parafilm was placed on top of the gelfoam. The skin was sutured and the animal was placed on a heating pad during recovery. In some cases, dura was labeled instead of MCA. In these animals, crystalline dye was placed on the dura in a region devoid of visible blood vessels. A piece of gelfoam saturated in dye was placed over the incision site and the wound was closed and the animal allowed to recover from anesthesia. During recovery, 5 µl of hydroxystilbamidine (Fluorogold, 10% solution in DMSO; Life Technologies) was slowly administered into the right nasal cavity of the rat to label trigeminal innervation of the nasal epithelium. After two weeks the animals were anesthetized and TGs harvested and processed for fluorescent microscopy as described previously. Images were acquired from nonadjacent tissue sections from each animal with a 10X objective lens and distance measurements made using Nis-Elements AR 3.0 (Nikon) software. Briefly, the distance between retrogradely labeled cells was measured from the center of the cell in question to the center of all other labeled cells in the image. Cells were included in measurements if their respective fluorescence intensity was 2-fold greater than their surrounding background intensity.

### Data collection and statistics

For blood flow experiments, data was collected at 1 Hz and filtered at 0.1 Hz for graphical representation. Basal blood flow was determined as the mean flow rate measured during a 4 minute period prior to drug application and the effects of test compounds were calculated by comparing the peak response after drug or saline administration to the basal blood flow proceeding administration. Changes in blood flow were calculated relative to the basal blood flow for each animal, averaged within treatment groups and expressed as percent changes. Comparison of blood flow changes in the absence of and following RTX injection was performed using a one way ANOVA followed by Tukey’s posthoc test. Comparison of TRPV1- and CGRP-immunoreactive cell counts was performed using a one way ANOVA followed by Tukey’s posthoc test. Retrograde labeled distance measurements obtained from 5 animals were exported to Excel (Microsoft) for analysis. qPCR results were calculated with the ΔΔC_T_ method as described [28]. qPCR data are presented as relative expression levels. Graphical presentation and statistical analysis was performed using GraphPad Prism 4 software (GraphPad, CA). Data values are presented as means ± SEM. The significance level for all tests was set at p<0.05.

## Results

### RTX administration depletes TRPV1 immunoreactive cells from trigeminal ganglia

**Figure 1:**
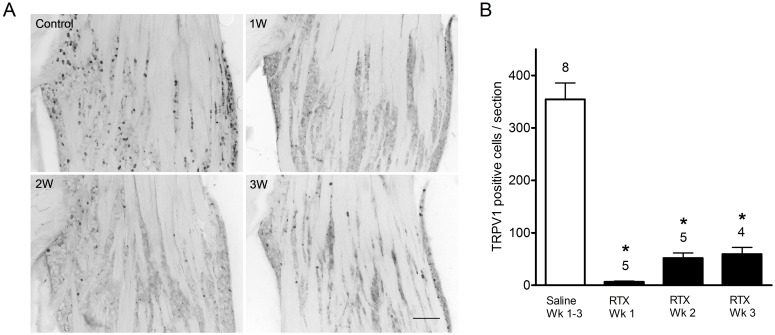
TRPV1 receptor immunoreactivity in trigeminal ganglia in vehicle- and RTX- treated rats. (A) TRPV1 receptor immunoreactivity is present throughout the ganglia in vehicle-treated but not in RTX-treated rats. One week following RTX treatment, TRPV1 receptor immunoreactivity is nearly abolished. After two and three-week survivals, TRPV1 receptor immunoreactivity is more evident but remains greatly diminished compared with vehicle-treated rats. Scale bar, 200 µm. (B) TRPV1 immunoreactive cell counts per section in the trigeminal ganglia in vehicle and RTX-treated rats. TRPV1 receptor cellular profiles are significantly decreased following RTX-treatment compared to vehicle treated at all survival times. Cell counts represented as mean ± S.E.M. Number of animals per group is indicated.

The ultrapotent TRPV1 receptor agonist RTX is known to selectively ablate capsaicin sensitive neurons in animals [30]. Here we have confirmed this effect and assessed the changes on TRPV1 immunoreactive cells in the TG. A single subcutaneous administration of RTX (200 µg/kg) dramatically decreased TRPV1 immunoreactivity in the TG. In vehicle injected control animals, TRPV1 immunoreactive cells were found to be homogenously distributed in the TG (Fig. 1A). Small to medium sized (20–50 µm diameter) TRPV1-positive cells were observed in V1, V2 and V3 divisions of the TG. One week following RTX administration, TRPV1 receptor immunoreactivity was nearly abolished in the TG. TRPV1-positive cell counts declined significantly from control values (354±31 vs. 6.6±1.5 cells; p<0.05) (Fig. 1B). TRPV1 receptor positive cell counts remained significantly depressed at two and three weeks of survival following RTX administration (51.8±10 and 59.2±13 cells respectively; p<0.05) when compared with control values. Similar neuronal loss following RTX treatment was observed with NeuN immunocytochemistry. Neuronal cell counts were significantly decreased following RTX treatment (351.0±29 cells; two and three week RTX survival pooled; n = 7) compared with control values (606.3±52 cells; n = 7: p<0.05). The number of total cell bodies lost is strikingly comparable to the decline of TRPV1 positive neurons, supporting the notion that RTX selectively ablates TRPV1 expressing neurons.

### RTX administration depletes CGRP immunoreactive cells from trigeminal ganglia

**Figure 2:**
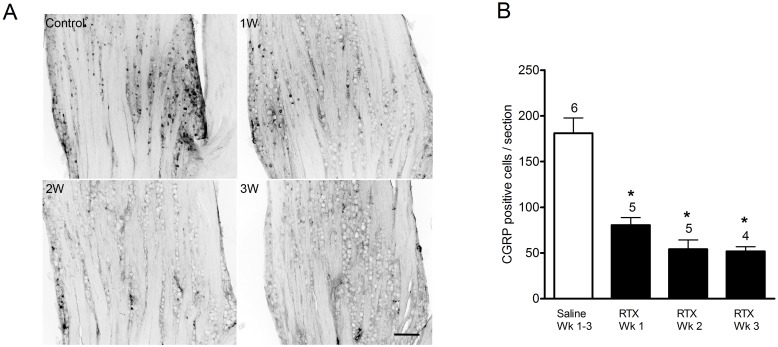
CGRP immunoreactivity in trigeminal ganglia in vehicle- and RTX- treated rats. (A) In vehicle-injected rats, CGRP immunoreactivity was evident in scattered small to medium sized neurons within the TG. RTX-treatment diminished CGRP immunoreactivity by 1 week of survival and further reduced positive profiles at two and three-week post RTX-treatment. Scale bar, 200 µm. (B) CGRP immunoreactive cell counts per section in the trigeminal ganglia in vehicle and RTX-treated rats. At all survival times (one, two and three weeks) following RTX treatment, CGRP positive cell counts are significantly reduced compared with vehicle-treated rats. Cell counts represented as mean ± S.E.M. Number of animals per group is indicated.

As one of the primary neurotransmitters released upon activation of sensory neurons, CGRP is thought to be an important mediator in migraine [31] and it has been shown that CGRP antagonists are efficacious in clinical trials [32]. In most [33], [34], but not all [35] reports, CGRP is highly co-localized with TRPV1 in the TG. Thus we examined whether RTX treatment also reduced the number of CGRP expressing cells in the TG. As seen in Fig. 2A, systemic administration of RTX induced a pronounced and long lasting reduction in CGRP immunoreactive cells in the TG. In vehicle-injected animals, small to medium sized CGRP -positive neurons were observed throughout the TG, particularly prominent in the V1 and V2 subdivisions. One week following RTX administration, a decrease in the number of CGRP -positive cells was observed which was confirmed by cell counts (Fig. 2B). CGRP-positive cells were significantly fewer after one week of RTX when compared with vehicle-injected animals (181±16.7 vs. 80.4±8.3 cells, p<0.05). Cell counts remained significantly decreased in the two- and three-week RTX-injected animals (54.2±10.2 and 51.7±5.1, respectively, p<0.05).

### RTX administration decreases TRPV1 and TRPA1 mRNA levels in trigeminal ganglia

**Figure 3:**
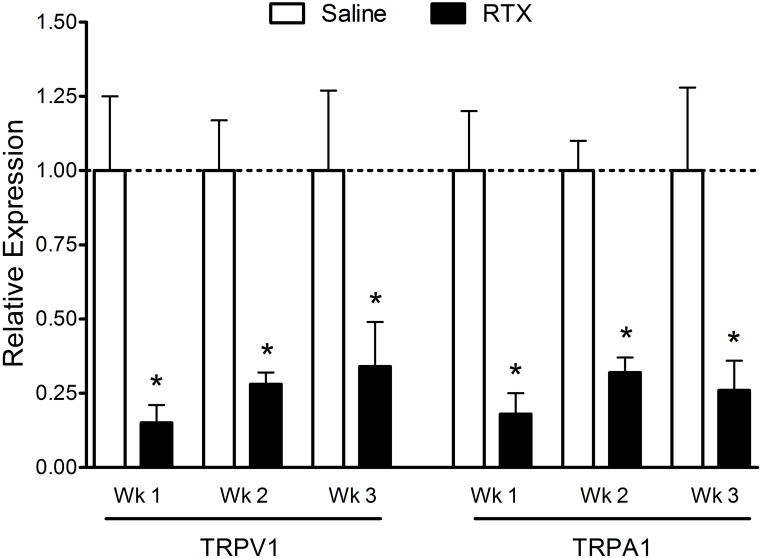
Relative expression levels of TRPV1 and TRPA1 mRNA in trigeminal ganglia following RTX treatment. RTX treatment induced a significant and similar reduction in both TRPV1 and TRPA1 mRNA expression levels at all survival times compared with vehicle injected controls. N = 4–9 animals per group.

Most reports indicate that TRPA1 receptors are co-expressed with TRPV1 receptors in sensory neurons [13], [14], [20], [21], [36], but other reports have demonstrated a population of cells sensitive to TRPA1 agonists but not to capsaicin [37], [38]. Therefore, we examined the effect of RTX administration on TRPV1 and TRPA1 mRNA expression in the TG. Consistent with our immunocytochemistry results, TRPV1 receptor mRNA levels were significantly decreased following RTX administration compared to vehicle-injected controls (Fig. 3). Compared to vehicle-injected controls, relative TRPV1 mRNA expression levels were significantly decreased (p<0.05) at one week (1±0.25 vs. 0.15±0.06), two weeks (1±0.17 vs. 0.28±0.04) and three weeks (1±0.27 vs. 0.34±0.15) post RTX treatment. Similarly, relative TRPA1 mRNA expression levels were reduced at one week (1±0.20 vs. 0.18±0.07), two weeks (1±0.10 vs. 0.32±0.05) and three weeks (1±0.28 vs. 0.26±0.10) post RTX treatment (p<0.05).

### RTX treatment blunts increases in meningeal blood flow induced by TRP agonists

**Figure 4:**
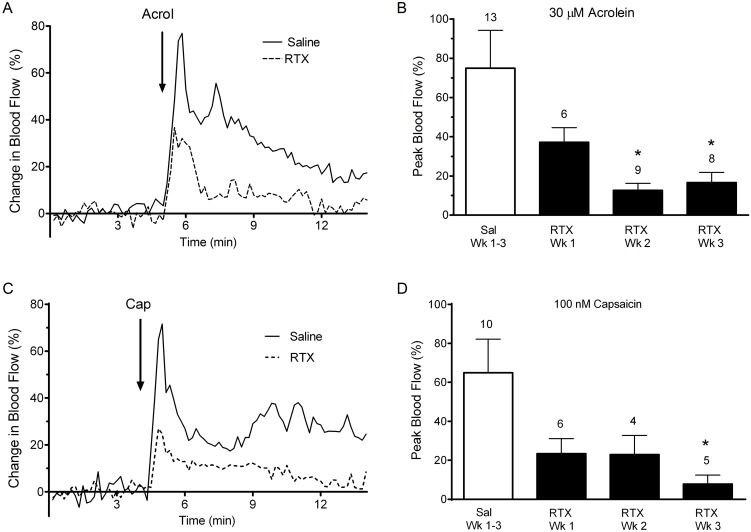
Blood flow changes in the middle meningeal artery following nasal administration of acrolein (A, B) or capsaicin (C, D) in RTX and vehicle injected animals. (A, C) Representative traces of middle meningeal blood flow changes in response to nasally administered acrolein or capsaicin. Laser Doppler flowmetry measurements were collected at 1 Hz and filtered at 0.1 Hz for graphical representation. Nasal application of acrolein or capsaicin in vehicle injected animals induced a rapid and robust increase in meningeal blood which returned toward baseline values within minutes while similar application of acrolein or capsaicin in RTX treated animals induced a reduced blood flow response. Arrows indicate nasal administration of agonist. (B, D) Compared to vehicle treated controls, blood flow response to nasal acrolein or capsaicin was diminished at one week of treatment and significantly decreased at two and three weeks of RTX treated. Similar diminished blood flow responses were observed with capsaicin in RTX treated animals. Values are means ± S.E.M. Number of animals per group is indicated. P<0.05 compared to blood flow changes in saline-injected animals.

We previously reported [8] that nasal administration of the TRPV1 receptor agonist, capsaicin, or the TRPA1 receptor agonist, acrolein, induced robust increases in meningeal blood flow in rats, suggestive of a role for these receptors in headache initiation. Furthermore, if these dural vasodilatory effects reflect activation of TRPV1/TRPA1 containing sensory neurons, RTX ablation of this population in the TG should attenuate this response. Here we examined this hypothesis by assessing the ability of TRPV1 and TRPA1 agonists to activate the nasal-meningeal pathway in RTX injected animals. Representative traces of MMA blood flow changes in response to nasal application of TRP agonists in control and RTX treated animals are shown in Fig. 4. Acrolein and capsaicin each induced a robust increase in blood flow in saline injected control animals (Fig. 4A and 4C, respectively). The blood flow changes were rapid, peaking within the first 1–2 minutes, and generally of short duration returning toward basal values within 10–15 minutes. In contrast, RTX pre-treatment attenuated the peak blood flow response seen with either TRP agonist (dashed lines, Fig. 4A and 4C).

The data summarizing TRP agonist induced blood flow changes from animals in the presence or absence of RTX pre-treatment are shown in Fig. 4B and 4D. Acrolein induced a peak blood flow change of 74.54±19.3% in the control group, while peak blood flow changes at one week following RTX treatment were diminished (37.17±7.4%) and significantly decreased by two and three week survivals (12.61±3.5% and 16.63±5.2%, respectively). Most of the animals (28/36) which were initially tested with acrolein were challenged with 1 µM capsaicin 15 min later. Peak blood flow changes in response to the capsaicin challenge paralleled the change in response to acrolein. Specifically, peak changes in blood flow in vehicle injected animals were 98.73±18.72%, and 21.5±9.01%, 11.52±0.59% and 8.94±4.33% in one, two and three week RTX treated animals, respectively. The blood flow reductions were significant in all RTX injected groups (p<0.05).

Similar blood flow changes following RTX treatment were observed when 100 nM capsaicin alone was tested (Fig. 4D). Peak changes in blood flow in vehicle injected animals were 64.9±17.27%, and 23.33±7.81%, 22.93±9.82% and 7.8±4.62% in one, two and three week RTX treated animals, with the three week reductions significant when compared to control. To assess whether RTX-injection might have compromised blood vessel function itself we examined responses to KCl applied to the dura (see Methods). Local application of KCl induced similar blood flow changes (20%) in both RTX and saline-injected animals demonstrating that RTX did not alter meningeal blood vessel responsivity per se (data not shown). Together these results demonstrate that RTX attenuates meningeal blood flow responses to nasal administration of TRPV1 and TRPA1 agonists and suggest that TRPA1 responses in the nasal-meningeal pathway are dependent upon functional TRPV1 expressing primary afferent neurons.

### Sensory neurons innervating the nasal epithelium vs the cranial vessels are in close mutual proximity within the trigeminal ganglion

Although TRPA1 receptors on trigeminal afferents in the nasal and respiratory epithelium are believed to be the initial sites of action of environmental irritants, it remains uncertain how this leads to blood vessel dilatation in the meninges, ostensibly innervated by a different population of TG sensory afferents. Trigeminal neurons innervating the nasal epithelium could conceivably propagate the signal to the meninges via trigeminal nerve collaterals as trigeminal neurons innervating the nasal cavity have collaterals projecting back to the cranium [39]. Alternatively, intraganglionic transmission [9], [40]–[42] could relay excitatory signals from nasal projecting neurons to nearby trigeminal neurons innervating the meninges. To discriminate these possibilities, we applied distinct retrograde dyes to the nasal epithelium and the MCA or dura and looked at the distribution of the two labels in the TG.

**Figure 5:**
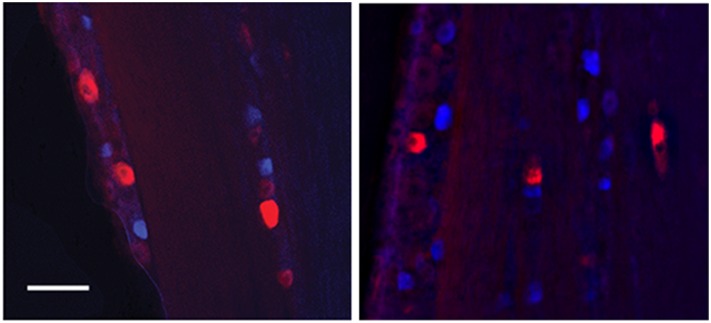
Retrograde double labeling in the trigeminal ganglion. Representative images two weeks following retrograde labeling of the nasal epithelium (Fluorogold; blue) and middle cerebral artery (MCA) (DiI; red). Numerous labeled cells were observed throughout the trigeminal ganglion and frequently retrograde labeled cells from the nasal mucosa were observed near to or adjacent to those from the MCA. Scale bar 100 µm.

Retrograde label from the nasal epithelium was most abundant in the dorsal half of the ipsilateral TG in V1 and V2 subdivisions consistent with previous reports [43], [44]. Small to medium sized labeled cells were distributed over the entire medial to lateral extent of the ipsilateral ganglion, though the highest density was localized along the dorsomedial border of the ganglion. As expected no labeled cell bodies were observed in the mandibular branch of the trigeminal nerve or in the contralateral ganglia. The pattern of retrograde labeling from the MCA and dura was similar to that observed from the nasal epithelium, but slightly more diffusely spread throughout the ganglion. Labeled neurons were prominent in the V1 and V2 subdivision of the ganglion, similar to previous reports [15], [29], though a small number of labeled neurons in the contralateral ganglia were also present. Co-localization of nasal and MCA/dural retrograde labels, an indicator of axonal collaterals supplying the distinct receptive fields, was not observed in any sections. In contrast, clusters of independently labeled neurons from each target were frequently observed to be clumped together (Fig. 5). Distance measurements between retrogradely labeled cells confirmed this observation (Table 1). The average nearest distance between individual Fluorogold (FG) nasal retrogradely labeled cells in the ganglia (n = 129) was 88.24±7.72 µm while that between retrogradely DiI labeled MCA cells (n = 110) was 120.90±12.05 µm. Distance measurements between FG labeled cells also revealed 37.2% of the cells (48 of 129 total labeled cells) were within 50 µm of the next closest FG labeled cell, and 70.5% of the total labeled population (91 of 129 cells) were within 100 µm. Slightly further distances were measured between nearest DiI labeled pairs, with 28.2% and 57.3% of the total population observed within 50 and 100 µm, respectively. The average nearest distance between a FG and DiI labeled cell was 95.01±8.98 µm, with distances similar to those for each separate population (24.8% and 69.8% of the total population observed within 50 and 100 um, respectively). The close proximity of these two afferent populations within the ganglia supports the potential involvement of chemical intraganglionic transmission to communicate signals from the nasal epithelium to the cranial blood vessels.

**Table 1:** Distance measurements between nasal epithelium sensory afferents (FG) and middle meningeal sensory afferents (DI) in the rat trigeminal ganglion.

Distance (µm)	FG→FG	%	DI→DI	%	FG→DI	%
0–50	48	37.2	31	28.2	32	24.8
51–100	43	33.3	32	29.1	58	45.0
101–150	26	20.1	19	17.3	25	19.4
151–200	8	6.2	19	17.3	6	4.6
201–250	3	2.3	3	2.7	2	1.5
251–300	0	0	5	4.5	3	2.3
301–350	0	0	1	0.9	2	1.5
351–400	0	0	0	0	1	0.7
401–500	1	0.8	0	0	0	0
N	129		110		129	

## Discussion

Headache is the most common symptom of air pollution, but few clinical studies have addressed the mostly anecdotal observations. Some studies have demonstrated that poor air quality is correlated with increases in ER visits due to headache [1]–[3], [45]. Headache, as well as other symptoms, are observed in Multiple Chemical Sensitivity, an acquired disorder believed to be caused by chemical exposure [46]. Usually these exposures are via inhalation, but how environmental irritants trigger headache is unknown. Excitation of trigeminal neurons results in the release of inflammatory mediators and meningeal vasodilatation. We hypothesized that inhalation of environmental irritants may excite trigeminal neurons supplying the nose and airways through TRPA1 receptors and subsequently cause trigeminovascular activation and headache [8]. TRPA1 is activated through covalent modification by a diverse array of structurally dissimilar chemicals including pungent plant compounds [14], [47], [48], products of oxidative stress [49]–[51] and environmental irritants [16], [17], [52]. Environmental irritants including acrolein and mustard oil activate TRPA1 resulting in sodium and calcium influx, increased intracellular calcium, neuronal excitation and neurotransmitter release from sensory neurons.

Activation and sensitization of the trigeminovascular system is believed to mediate headache pain [53]. Animal models of migraine often assess neurogenic inflammation of the meninges as a marker for activation of the trigeminovascular system [54]. Although the clinical correlation between vasodilatation and headache are unclear, assays of meningeal vasodilatation are widely used in migraine models and have proven to be predictive of clinical efficacy. Using laser Doppler flow measurements of blood flow in the meninges we previously demonstrated that nasal administration of environmental irritants can activate the trigeminovascular system via TRPA1 receptors [8]. Nasal administration of acrolein and mustard oil induced meningeal vasodilatation, which was blocked by prior nasal application of a TRPA1 antagonist suggesting a critical role for TRPA1 in environmental irritant-induced headache. This concept has been corroborated by recent studies identifying umbellulone, the active ingredient of the “headache tree”, as a TRPA1 agonist [18], [55]. Individuals experience headache after exposure to fumes of the California bay laurel or Umbellularia californica tree [56]. Umbellulone, the most abundant volatile component from the leaves of the headache tree, was found to be a TRPA1 agonist capable of eliciting meningeal blood flow after nasal administration and CGRP release when applied to trigeminal neurons [18], [55].

While these studies suggest that nasal administration of TRPA1 agonists stimulate the trigeminovascular system, the specific sites associated with TRPA1 action have not been determined. TRPA1 agonists could conceivably elicit meningeal vasodilatation either by acting locally in the nasal mucosa or via the blood stream to act in the meninges or at yet other sites. Herein we have begun to explore the anatomical pathway involved and the function of TRP channels in this pathway.

Multiple members of the TRP channel family are expressed in the TG, including TRPV1 and TRPA1 both of which have been implicated in nociception. Evidence supports the co-expression of TRPA1 and TRPV1 in a subset of sensory neurons [13], [14], [20], [21], [36], while some neurons appear to only express TRPA1 [37], [38]. The TG contains a heterogeneous population of cells where relative expression patterns of TRP channel family members may define distinct functional subsets of sensory neurons [38], [57]. Only limited studies [15] have examined co-localization of TRPV1 and TRPA1 in sensory neurons or pathways so the functional significance of co-expression is unclear. When TRPV1 and TRPA1 are co-localized they can interact to modulate function in sensory neurons [22]. Therefore we examined the importance of TRPV1-TRPA1 co-expression in the mechanism of nasal-meningeal vasodilatation.

Administration of RTX, an ultrapotent TRPV1 agonist [30], [58] can reduce TRPV1 receptor binding, selectively ablates the function of TRPV1 expressing neurons [59], [60] and is currently in use clinically for pain relief [61], [62]. In adult rats RTX treatment attenuates multiple types of pain behaviors, including loss of sensitivity to thermal and chemical stimuli [24], [63], [64], while mechanonociception, other sensory modalities and motor function are maintained [63]. Consistent with previous reports [63], [65], [66], we observed significantly reduced TRPV1 immunoreactivity throughout the TG following a single RTX injection compared to saline injected controls. Nearly identical loss of NeuN immunoreactive cells and TRPV1 immunoreactive cells in adjacent sections supports our conclusion that RTX selectively ablated TRPV1 expressing cells. In addition to reduced TRPV1 expression, CGRP immunoreactivity was also significantly decreased in the TG after RTX injection. RTX had a similar effect on CGRP-immunoreactive neurons in dorsal root ganglia [67] and the combined loss of expression is consistent with observations of co-localization of TRPV1 and CGRP in primary sensory neurons [14], [33]–[35], [68].

TRPV1 and TRPA1 mRNA levels were also found to be significantly decreased in the TG of RTX injected animals. RTX injected rats displayed a significant and sustained reduction in mRNA expression for both receptors, a reduction similarly observed in rat dorsal root ganglion [64]. Our results are also consistent with previous studies showing significantly decreased protein levels of both receptors following RTX administration [66], [69]. However others [70] have reported that RTX treatment reduced TRPV1-immunoreactivity but had little effect on TRPA1-immunoreactive neurons in mouse TG. Mishra and Hoon [70] observed nearly a 70% reduction in TRPV1 immunoreactive neurons and decreased sensitivity to capsaicin following RTX treatment, but no effect on the number of TRPA1-immunoreactive neurons or sensitivity to mustard oil. They further found a population of large diameter TRPA1 and TRPV1 co-expressing neurons in the TG following RTX treatment and postulated that the large neuronal volume may make this subset of neurons more resistant to RTX. While we also observed a significantly reduced population of TRPV1 immunoreactive cells and mRNA expression following RTX treatment, the uniform decrease of TRPV1 and TRPA1 mRNA expression levels reported here argues against a RTX-resistant TRPA1^+^ cell population and supports the reports of a nearly complete co-localization of TRPA1 in TRPV1 expressing primary sensory neurons [13], [14], [36]. Differences between the studies may be due to the dosage, route of administration or species of the animals. Furthermore, after an extensive search we failed to identify a reliable and specific TRPA1 antiserum of high titer for our use, thus we elected to measure TRPA1 mRNA. Thus there may be concerns with some reagents used in previous studies of TRPA1 protein expression. Overall, these results demonstrate that RTX ablated TRPV1 expressing cells and that both CGRP and TRPA1 are highly co-localized with TRPV1 in trigeminal ganglion neurons.

In a previous study, we showed that prior nasal application of TRPV1 or TRPA1 antagonists significantly reduced agonist induced meningeal vasodilatation [8]. Here, RTX treatment significantly reduced meningeal blood flow stimulated by nasal administration of TRPV1 and TRPA1 agonists. The reductions of MMA blood flow, TRPV1 and CGRP immunoreactivity, and TRPV1 and TRPA1 mRNA expression, are all consistent with the hypothesis that TRPA1 receptors, co-expressed with TRPV1, play a critical role in environmental irritant-induced headache [8], [18], [55]. Surprisingly, MMA vasodilatation upon administration of either agonist was not significantly diminished in the one-week RTX treated animals. One possible explanation for this temporal discrepancy may be that RTX is known to produce paradoxical changes in thermal and mechanical sensitivities which have been attributed to abnormal sprouting of afferent fibers [24] and/or upregulation of peripheral receptors [67]. Another possibility may be a difference in the effect of RTX on receptors in the central spinal TG versus peripheral receptors lining the nasal epithelium, as studies have shown differential effects between central and peripheral TRPV1 receptors following RTX administration [63], [71]. Furthermore, it is noteworthy that capsaicin and other TRPV1 agonists have shown some efficacy in migraine and cluster headache after peripheral administration (for review, see Oxford and Hurley, 2013 [72]), using methods which are likely to inhibit only a small subpopulation of TRPV1 expressing neurons.

The anatomical pathway of nasal-meningeal signaling is not well-understood. Several mechanisms have been proposed to explain neurogenic inflammation at a peripheral site distant to the initial site of stimulus, including axon reflex and intraganglionic transmission. We therefore employed retrograde labeling of nasal and dural afferents to gain some insight into their ganglionic organization. We observed no co-localization of labels, but rather that nasal and cranial labeled cells were located in close proximity to each other within the TG. To our knowledge this is the first report that has examined this question and to describe a close spatial relationship between TG neurons that innervate the nose and cranial vessels. Our results are similar to previous reports of a close association in the TG of dural afferents with sensory afferents from the forehead and periorbital facial region [15], [29]. Nearest neighbor analysis revealed that soma of sensory afferents from the face are clustered around cranial arterial afferent soma in the TG, and that forehead cells are also clustered with each other [29]. Our labeling data suggest that an “axon reflex” [73] is unlikely to mediate the nasal-meningeal pathway of environmental irritant-induced headache and, instead, points toward the possibility that intraganglionic transmission may have a role. A recent study by Huang and colleagues [15] reached a similar conclusion based on the close proximity of dural afferents with TRPA1 positive neurons in the mouse TG. They estimated one neuron on average separated each TRPA1 neuron from its nearest dural afferent. Likewise we observed nearly 25% of nasal epithelium afferents within 50 µm of a labeled cranial afferent and over 70% within 100 µm. This close proximity suggests the potential that neurotransmitter and/or neuropeptide released from nasal epithelium afferents would have a high likelihood of cross-exciting cranial afferents. Cross-excitation and somatic neurotransmitter release has been observed in sensory ganglion [74] [9], [10], [42] and has been proposed as the mechanism for the referred pain of migraine [29], [75].

In summary, our data demonstrate that neurons expressing functional TRPV1 are necessary for TRPA1 responses in the nasal-meningeal pathway. Our data are consistent with a role for intraganglionic transmission in the nasal-meningeal pathway and suggest that nasal-meningeal signaling is not primarily by axon reflex, but we cannot rule out other mechanisms at this time. A more complete understanding of this pathway awaits further studies.
